# *In Vitro* Assessment of Clevidipine Using the Profilin1 Hypertensive Mouse Model

**DOI:** 10.3390/ph6050623

**Published:** 2013-04-29

**Authors:** Hamdy H. Hassanain, Mohamed D. H. Hassona, Erika G. Puente, Chengwen Sun, Zeinb A. Abouelnaga, David B. Tulman, Sergio D. Bergese

**Affiliations:** 1 Molecular, Cellular and Developmental Biology Program, the Ohio State University Wexner Medical Center, 1645 Neil Avenue, Columbus, OH 43210, USA; E-Mail: Sergio.Bergese@osumc.edu; 2 Department of Anesthesiology, the Ohio State University Wexner Medical Center, 410 W. 10th Ave, Columbus, OH 43210, USA; E-Mails: mohdhessein@yahoo.com (M.D.H.H.); Erika.Puente@osumc.edu (E.G.P.); Zeinb.Abouelnaga@osumc.edu (Z.A.A.); David.Tulman@osumc.edu (D.B.T.); 3 Department of Pharmaceutical Science, North Dakota State University, 1401 Albrecht Blvd., Fargo, ND 58102, USA; E-Mail: Chengwen.Sun@ndsu.edu; 4 Department of Neurosurgery, the Ohio State University Wexner Medical Center, 410 W. 10th Ave, Columbus, OH 43210, USA

**Keywords:** clevidipine, transgenic profilin1 mice, vascular hypertrophy, wire-myograph

## Abstract

Hypertension represents a major risk factor for cardiovascular events, associating with vascular hypertrophy and dysfunction in resistance vessels. Clevidipine is a novel antihypertensive drug working as a selective calcium channel antagonist with an ultra-short half-life that lowers arterial blood pressure by reducing systemic arterial resistance. The aim was to assess the effect of clevidipine on the hypertrophic vessels of profilin1 hypertensive transgenic mice compared to sodium nitroprusside (SNP) and labetalol using wire myograph techniques. The effects of clevidipine, SNP and labetalol on the hypertrophic vessels were studied on mesenteric arterial function from 8 profilin1 hypertrophic mice and eight non-transgenic controls. Our results showed a significant difference between the effects of the three drugs on the hypertrophic mesenteric arteries of transgenic profilin1 mice compared to the non-transgenic controls. The half maximal effective concentration (EC_50_) of clevidipine, SNP and labetalol in profilin1 mice (1.90 ± 0.05, 0.97 ± 0.07, 2.80 ± 0.05 nM, respectively) were significantly higher than the EC_50_ in non-transgenic controls (0.91 ± 0.06, 0.32 ± 0.06, 0.80 ± 0.09 nM, respectively). Moreover, the increase in the EC_50_ for clevidipine (2-fold) to produce the same effect on both normal and hypertrophic arteries was less than that of SNP (3-fold) and labetalol (3.5-fold). Therefore, we concluded clevidipine exhibited the lowest dose shift to relax the hypertrophic vessels compared to SNP and labetalol in the profilin1 hypertrophic animal mouse model.

## 1. Introduction

Hypertension represents a major risk factor for cardiovascular events such as stroke and myocardial infarction. According to American Heart Association estimates, hypertension affects some 76.4 million adults (33.5% of the adult population) and is a major risk factor for mortality [1]. The management of acute hypertension in some clinical circumstances requires the immediate reduction of the mean arterial pressure (MAP), which necessitates the use of intravenous antihypertensive agents. The ideal antihypertensive has been described as: (1) able to predictably control blood pressure (BP), (2) have a good safety profile and (3) a rapid onset and offset of effect. Labetolol and sodium nitroprusside (SNP) are frequently used antihypertensive agents [2]. SNP has long been considered the standard against which other intravenous antihypertensive agents are compared [3,4]. SNP is a direct-acting, potent nitrovasodilator that affects both the venous and arterial vasculature. It can be converted into nitric oxide, a smooth muscle relaxing factor synthesized by the eNOS gene [5]. Labetalol is a racemic mixture of four diastereomers that have unique effects at α1- and β-adrenergic receptors, with a net effect of α1-receptor and nonselective β-receptor antagonism [6]. Patients with hypertensive urgencies/emergencies require rapid and controlled lowering of MAP, and may benefit from the use of antihypertensive agents that have rapid onset and offset of action and are easily titratable.

Lowering BP and treating hypertension are two different concepts. Many drugs lower BP; however, only a few are effective in controlling hypertension in a precise manner [7]. This has led to the introduction of new intravenous agents such as nicardipine (second generation) and clevidipine (third generation). Clevidipine is a dihydropyridine calcium channel antagonist that is available as a racemic mixture in a lipid emulsion for intravenous infusion. Clevidipine is a rapidly-acting and predictable, vascular-and arterial-selective dihydropyridine L-type calcium antagonist with a half-life of one minute that lowers arterial MAP by reducing systemic arterial resistance. It exerts a selective vasodilator action on arteriolar resistance vessels, but has no effect on venous capacitance vessels. Due to its fast onset and offset of effect on MAP, clevidipine can easily be titrated, allowing for rapid and controlled management of BP [8]. Resistance vessels are known to play an important role in regulating MAP. These vessels include small arteries and arterioles, with diameters ranging from 150–300 µm [9,10]. Furthermore, most acute hypertensive patients suffer from chronic hypertension which often associates with vascular hypertrophy [11]. Thus, the focus of this report is to assess the relaxing effect of clevidipine on resistant arteries with hypertension-associated vascular hypertrophy from profilin1 transgenic mice, compared to other commonly used antihypertensive drugs, such as SNP and labetalol.

The profilin1 transgenic mouse model was developed in our laboratory by transgenically overexpressing the cDNA of the human profilin1 gene in vascular smooth muscle cells (VSMCs) using the mouse VSM α-actin promoter [12]. Our previous study showed that overexpression of profilin1cDNA in VSMCs of the transgenic mice leads to vascular hypertrophy in the aortas and mesenteric arteries as well as elevation of BP when the mice reach 6 months of age [12,13].

## 2. Experimental Section

### 2.1. Animals

Mice were bred and maintained at the W.M. Keck Genetic Research Facility of The Ohio State University (OSU, Columbus, OH, USA), and the experimental procedures in mice and protocol used in this study were approved by the Animal Care and Use Committee of OSU, conforming with the Guide for Care and Use of Laboratory Animals published by the U.S. National Institutes of Health (NIH publication No. 85-23, revised 1996). For this study, we used male profilin1 transgenic mice and age-matched non-transgenic controls. The construct and the procedure for the generation and characterization of the profilin1 transgenic mice have been described before [12]. This model was developed in our laboratory by transgenically overexpressing the cDNA of the human profilin1 gene in VSMCs using the mouse vascular smooth muscle a-actin promoter. The profilin1 transgenic mice model shows a MAP significantly higher than that of non-transgenic controls [12,13,14]. Evidence has also demonstrated that histological changes show signs of remodeling and vascular hypertrophy in the aortas and mesenteric arteries from the profilin1 transgenic mice as compared to non-transgenic controls [12,13]. Unless otherwise stated, all the experiments were performed with heterozygous transgenic profilin1 mice (8- to 12-months-old) and with age-matched non-transgenic littermates. Eight mice per genotype were used for each experiment.

### 2.2. Vascular Reactivity

Mice were anesthetized with pentobarbital sodium, and the mesentery was rapidly excised and placed in an ice-cold 4-(2-hydroxyethyl)-1-piperazineethanesulfonic acid (HEPES)-physiological saline solution (PSS). The composition of HEPES-PSS was as follows (in mM): 142 NaCl, 4.7 KCl, 1.17 MgSO_4_, 1.56 Ca_2_Cl, 1.18 KH_2_PO4, 10 HEPES, and 5.5 glucose. Second-order branches of mesenteric artery (2 mm in length with internal diameter 150 to 200 µm) were carefully dissected and mounted as ring preparations on two stainless steel wires (40 µm diameter) as described in our previous reports [13,14]. The second-order mesenteric arteries were mounted in an isometric Mulvany-Halpern small vessel myograph (Model 610 M, Danish Myo Technology, Aahrus, Denmark), and data were acquired by a PowerLab 8SP data acquisition system (AD Instruments, Colorado Springs, CO, USA). A resting tension of 3 mN was imposed on the second-order mesenteric arteries, and vessels were equilibrated for 1 hour in HEPES-PSS at 37 °C and continuously bubbled with 5% CO_2_ and 95% O_2_. The 3 mN is used as the arbitrary resting tension based on our preliminary studies. For our mesenteric arteries prepared from mice, the 3 mN vascular tone is equivalent with 90 mmHg, which is the normal basal blood pressure in mice [15]. Arterial integrity was assessed first by stimulation of vessels with 80 mM KCl. Endothelium integrity was assessed by measuring the dilatory response to acetylcholine (ACh) (1µM) in phenylephrine-contracted vessels (1 µM). The failure of ACh to relax denuded mesenteric rings was considered proof of endothelium disruption. The dilatory responses of each drug, clevidipine, SNP and labetalol, were then assessed on profilin1 hypertrophic mesenteric arteries pre-constricted with phenylephrine (1 μM) and compared to normal mesenteric arteries from matching control littermates. The vascular dilator response to the drugs was expressed as the percentage of basal tone induced by phenylephrine (1 µM).

### 2.3. Statistical Analysis

Data are presented as mean ± S.E. Data were analyzed by unpaired t test. A two tailed value of *p* < 0.05 was considered significant as compared to control unless otherwise stated.

## 3. Results and Discussion

### 3.1. Effect of Clevidipine on Phenylephrine-Contracted Profilin1 Mesenteric Arteries

**Figure 1:**
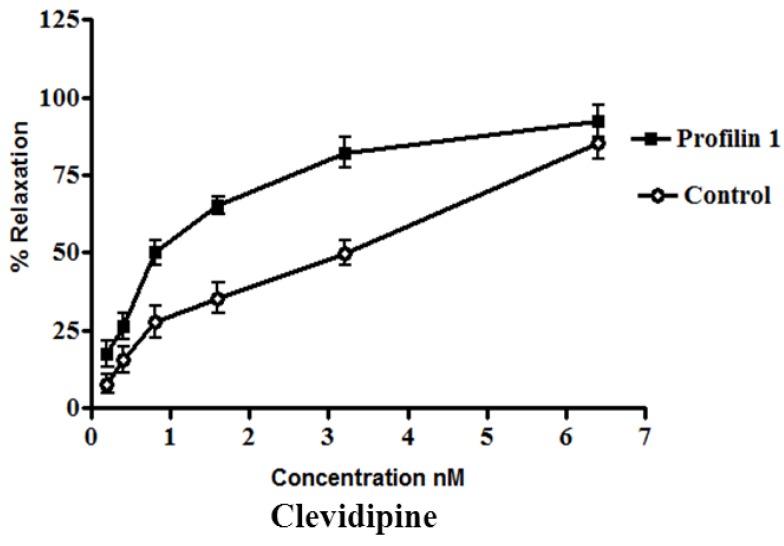
Effect of clevidipine on phenylephrine-contracted profilin1 mesenteric arteries. Vascular reactivity of profilin1 and control mesenteric arteries (n = 8) was assessed towards clevidipine at different concentrations using the myograph system. The results show adecrease in the relaxing responses in profilin1 mesenteric arteries compared to non-transgenic controls.

The dilator response of clevidipine was tested in the mesenteric arteries from profilin1 transgenic mice and controls. The mesenteric arteries were pre-constricted with phenylephrine (1 µM). Phenylephrine-induced vascular basal tone in arteries from profilin1 transgenic hypertensive mice was enhanced compared with control mice (17.8 ± 1.1 mN and 12.4 ± 0.9 mN respectively, n = 8, *p* < 0.05). The phenylephrine pre-constricted mesenteric arteries were concentration dependently relaxed by clevidipine (Figure 1) with an increase in half maximal effective concentration (EC_50_) of profilin1 transgenic mice (1.9 ± 0.052 nM) two times as EC_50_ of non-transgenic control (0.91 ± 0.059 nM).

Clevidipine alone (0.1 nM–0.1 µM.) had no effect on the basal tension of mouse mesenteric arteries. Results showed a significant decrease in the vessel relaxation response of profilin1 mesenteric arteries toward clevidipine compared to non-transgenic controls (*p* < 0.05) (Figure 1).

### 3.2. Effect of SNP on Phenylephrine-Contracted Profilin1 Mesenteric Arteries

**Figure 2:**
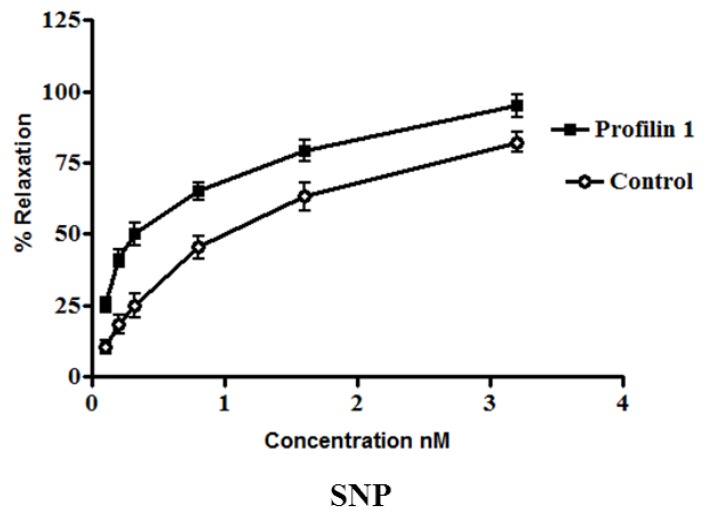
The effects of sodium nitroprusside (SNP) on vascular reactivity of profilin1 mesenteric arteries. Vascular reactivity in profilin1 and control mesenteric arteries (n = 8) was assessed towards SNP at different concentrations using the myograph system. The results show a decrease in the relaxing responses in profilin1 mesenteric arteries compared to non-transgenic controls.

The dilator response of SNP was examined in the mesenteric arteries from profilin1 transgenic mice and controls using the same protocol as mentioned for the clevidipine group. Phenylephrine-induced vascular basal tone in arteries from profilin1 transgenic hypertensive mice was enhanced compared with control mice (14.8 ± 1.3 mN and 12.8 ± 1.1 mN respectively, n = 8, *p* < 0.05). SNP treatment induced a dose-dependent dilation in both hypertrophic arteries and controls (Figure 2). However, SNP-induced vasodilation is significantly decreased in hypertrophic arteries as compared with controls. The results showed that phenylephrine pre-constricted mesenteric artery tissues were concentration-dependently relaxed by SNP with an increase in EC_50_ of profilin1 transgenic mice (0.97 ± 0.072 nM) three times as EC_50_ of non-transgenic control (0.32 ± 0.061 nM). SNP alone (0.1 nM–0.1 µM.) had no effect on the basal tension of mouse mesenteric arteries. Results showed a significant decrease in the vessel relaxation response of profilin1 mesenteric arteries compared to non-transgenic controls (*p* < 0.05) (Figure 2).

### 3.3. Effect of Labetalol on Phenylephrine-Contracted Profilin1 Mesenteric Arteries

**Figure 3:**
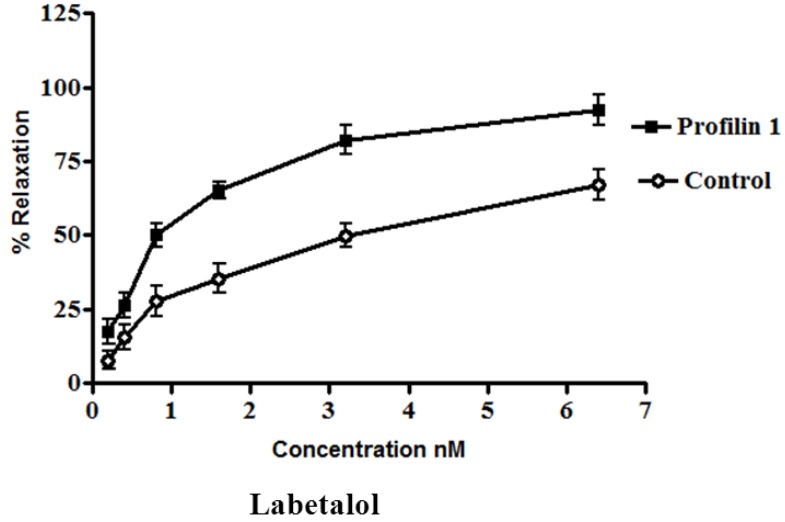
The effects of labetalol on vascular reactivity of profilin1 mesenteric arteries. Vascular reactivity of profilin1 and control mesenteric arteries (n = 8) was assessed towards labetalol at different concentrations using the myograph system. The results show adecrease in the relaxing responses in profilin1 mesenteric arteries compared to non-transgenic controls.

The dilator response of labetalol was determined in the mesenteric arteries from profilin1 transgenic mice and controls using the same protocol as mentioned before. Phenylephrine-induced vascular basal tone in arteries from profilin1 transgenic hypertensive mice were enhanced compared with control mice (14.1 ± 1.0 mN and 11.9 ± 1.3 mN respectively, n = 8, *p* < 0.05). The phenylephrine pre-constricted mesenteric artery tissue was concentration- dependently relaxed by labetalol with an increase in EC_50_ nM of profilin1 transgenic mice (2.8 ± 0.045 nM) 3.5 times as EC_50_ of non-transgenic control (0.80 ± 0.093 nM). Labetalol (0.1 nM–0.1µM.) alone had no effect on the basal tension of mouse mesenteric arteries. Results showed a significant decrease in the vessel relaxation response of profilin1 mesenteric arteries compared to non-transgenic controls (*p* < 0.05) (Figure 3).

### 3.4. Comparative Analysis of the Effects of Clevidipine, SNP and Labetalol on Phenylephrine-Contracted Hypertrophied Profilin1 Mesenteric Arteries

**Figure 4:**
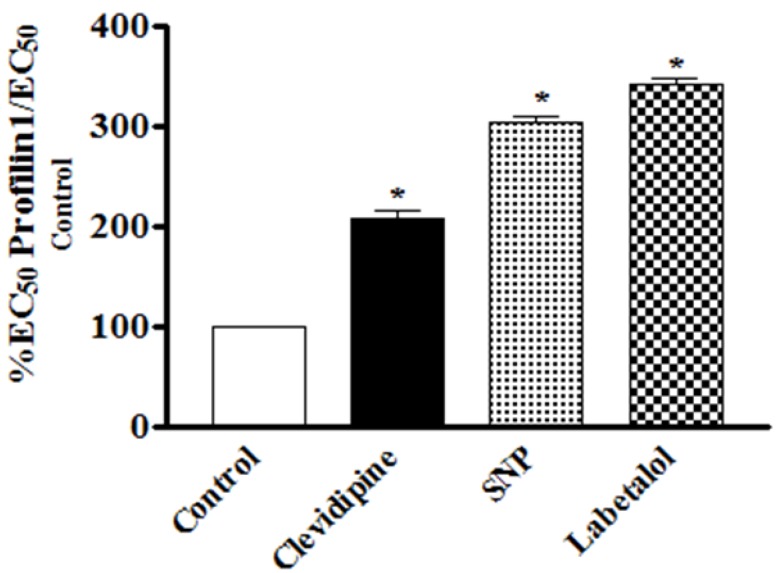
Comparative analysis of the effects of clevidipine, SNP and labetalol on phenylephrine-pre-contracted hypertrophied profilin1 mesenteric arteries. There is a significant difference in the effect of clevidipine compared to SNP and labetalol. While we need only to increase the clevidipine EC50 two fold in the profilin1 to obtain the same degree of relaxation of its control, the SNP and labetalol needed to be increased 3 and 3.4 times respectively to obtain the same relaxing effects as seen in their controls.

In this study, the vasodilating potency and efficacy of clevidipine have been assessed on contracted vessels of profilin1 mice as compared with SNP and labetalol. At a dose range of 0.2–3.2 nM clevidipine showed a less potent relaxant effect at all doses compared to SNP, yet, it did not reach significance. However, when looking at the EC_50_, the higher potency of SNP becomes very clear: The EC_50_ of SNP (0.97 ± 0.072 nM) to relax phenylephrine-pre-contracted hypertrophied profilin1 mesenteric arteries was significantly lower than that of clevidipine (1.9 ± 0.052 nM) (Table 1). Conversely, at the highest dose (3.2 nM) both drugs demonstrated a similar maximal effect signifying that both clevidipine and SNP exhibited comparable efficacy in our model. At the same dose range clevidipine displayed a more potent relaxant effect at all doses compared to labetolol. This was clearly significant at the highest drug dose (3.2 nM), but not at the other lower investigated doses (Figure 4). However, when looking at the EC_50_, the higher potency of clevidipine becomes very clear: The EC_50_ of clevidipine (1.9 ± 0.052 nM) to relax phenylephrine-pre-contracted hypertrophied profilin1 mesenteric arteries was significantly lower than that of labetolol (2.8 ± 0.045 nM) (Table 1). Additionally, clevidipine exhibited higher efficacy compared to labetolol in our model as indicated by the highly significant maximal response obtained by clevidipine at the highest dose.

**Table 1:** The EC_50_ of clevidipine, sodium nitroprusside (SNP) and labetolol in control and profilin1 mesentric arteries. There is a significant difference in the effect of clevidipine compared to SNP and labetalol. Data are presented as means ± S.E, (n = 8).

Drug	EC_50_ (nM)		EC_50_ (Fold increase)
	Control	Profilin	
Clevidipin	0.91 ± 0.059 ^#^	1.9 ± 0.052 *^,+^	2.1
SNP	0.32 ± 0.061	0.97 ± 0.072 *	3
Labetolo	0.80 ± 0.093	2.8 ± 0.045 *^,+,^^	3.5

*, indicates significant difference compared to corresponding EC_50_ in control vessels (unpaired t test); ^#^, indicates significant difference compared to EC_50_ of SNP in control vessels (ANOVA); ^+,^^ indicate significant differences compared to EC_50_ of SNP and clevidipine in hypertrophied profilin1 vessels, respectively, (ANOVA).

Interestingly, our results also showed that there was a need to increase the EC_50_ of clevidipine up to 200% compared to the normal control vessels to obtain the same relaxing effect (50% relaxation) on the profilin1 hypertrophic vessels (Figure 4). However, the need to increase the EC_50_ from normal vessels with SNP or labetalol was 300% and 350%, respectively, to reach 50% relaxation in the hypertrophic vessels as seen in their control vessels (Table 1).

### 3.5. Discussion

Initial intravenous antihypertensive therapies used to control BP vary. Although several intravenous antihypertensive agents are currently available, including SNP, nitroglycerine, nicardipine, and labetalol, most have limitations due to their pharmacologic properties. The choice of a certain agent will depend on the clinical presentation and pharmacological properties of the agent. The STAT registry recently demonstrated that 64% of the patients required multiple drugs to achieve an effective decrease in BP. The most common treatment agent used to treat acute hypertension was labetalol (32%), an antihypertensive agent with a unique mode of action as an α- and β-adrenoreceptor antagonist that lowers BP by blocking α-adrenoreceptors in the peripheral arterioles and lowering peripheral vascular resistance. The effectiveness of labetalol to lower BP has proven to be disappointing and quite unpredictable as the failure rate from the STAT registry showed that 32% of the patients required one dose, 42% required two doses, and 25% required three or more doses to achieve the desired BP decrease [16,17]. Other IV antihypertensive drugs such as nitroglycerine and SNP are short-acting vasodilators commonly used in the management of acute hypertension. However, these drugs have disadvantages such as tachyphylaxis, reflex tachycardia and rebound hypertension. Another disadvantage of both is the lack of vascular selectivity, causing not only arterial dilatation, but venous dilatation as well [18]. Studies on SNP also mention side effects such as cyanide toxicity, which was initially demonstrated in animal studies [19,20]. Other drugs from the dyhidropyridine calcium channel antagonist group such as nifedipine and nicardipine different to nitrovasodilators have a relatively longer plasma half-life and extended duration of effect. These limitations have prompted the development of new intravenous drugs such as clevidipine. In contrast to these current vasodilators that act through α-and-β-receptor antagonists, clevidipine is a late-generation dihydropyridine calcium channel vasodilator that decreases systemic vascular resistance without affecting venous capacitance vessels [21].

In our previous study [12], we demonstrated that increased expression of human cDNA of profilin1 in VSMCs of transgenic mice resulted in alteration of cytoskeleton dynamic favoring increased actin polymerization that can lead to hypertrophy in blood vessels and elevation in MAP when the mice reach the age of 6-months-old [12,13]. The Rho protein, through its downstream effector Rho-associated protein kinase (ROCK), mediates cytoskeletal reorganization as well as smooth muscle cell contraction and is implicated in the pathophysiology of hypertension. Spontaneously hypertensive rats have demonstrated a significant increase in Rho expression and activity [22]. The profilin1 transgenic mouse is a good model that allowed us to evaluate the effects of different antihypertensive drugs in the hypertrophic vessel [14], simulating those of chronic hypertensive patients. Essential hypertension in humans is often associated with vascular hypertrophy [23]. Essential hypertensive patients constitute a majority the patients with cardiovascular disease who are susceptible to hypertensive crisis [24]. In fact, clevidipine has been studied before in patients with essential hypertension, preoperative treatment of hypertension and other clinical trials. Clevidipine showed better preservation in target blood pressure range than other antihypertensive drugs such as nitroglycerine and SNP, and it was generally well tolerated [20,25]. Therefore it is beneficial to reproduce and determine the effects of drugs used in the treatment and management of acute hypertension such as clevidipine, SNP and labetalol in an animal model.

Our results showed a significant decrease in the relaxation response of profilin1 mesenteric arteries towards clevidipine, SNP and labetalol compared to non-transgenic control arteries. The clevidipine, SNP and labetalol concentrations that were required to inhibit the contracted profilin1 mesenteric arteries were higher than those concentrations required for controls due to the significant vascular hypertrophy present in the profilin1 mesenteric arteries. Our results are in agreement with other reports that showed that the structural remodeling of small blood vessels alter the response to vasoactive agents, as shown in hypertensive patients [23,26]. In addition, we showed that clevidipine exhibited comparable efficacy to that of SNP and a higher efficacy than that of labetolol as a vasodilator when used in hypertensive mice with hypertrophied blood vessels such as profilin1 mice. Importantly, our results showed that the concentration of clevidipine required to achieve the same effect was only twice as much as the concentration required in normal vessels, while the concentrations of SNP and labetalol were required to be increased three and three and half times, respectively, to reach the same levels of relaxation as those of the controls. This may impact the use and increase the safety profile of clevidipine compared to the two other drugs, especially SNP, which could cause lethal side effects at high doses [19,20].

In the current experiment, we assessed the effect of clevidipine on the phenylephrine-pre-constricted mesenteric arteries of profilin1 transgenic mice and normal controls. Our data demonstrated that clevidipine cause dose-dependent vasodilator response. This result is consistent with a previous publication showing that clevidipine is a potent arterial dilator in human internal mammary artery [27]. Phenylephrine is a selective agonist of α1-adrenergic receptor, which is a G-protein coupled receptor, localized on the VSMCs. Activation of this receptor stimulates L-type calcium channel via several intracellular signaling pathways. Calcium influx through L-type calcium channel triggers calcium-induced calcium release from intracellular store, leading to further increased intracellular calcium elevation, inducing VSM constriction. Clevidipine is a calcium channel blocker, which shut down L-type calcium channel, leading to relaxation of VSM pre-constricted by phenylephrine. In addition, we also compared the vasodilator effect of clevidipine in mesenteric arteries of profilin1 transgenic mice with that of normal control mice, indicating that the vasodilator response to clevidipine is decreased in profilin1 transgenic mice. The molecular mechanisms under this phenomenon are still not clear. Our previous study and the current study demonstrate that profilin1 transgenic mice develop vascular hypertrophy and hypertension, which lead to vascular remodeling and stress fiber formation. All of these pathophysiological alterations induce the stiffness of the vascular wall, which diminish vasodilator response to clevidipine.

Here, we also compared the vasodilator response of clevidipine with other commonly used drugs, SNP and labetalol. Their vasodilator effects are all decreased in hypertrophic arteries as compared with controls. However, the clevidipine-induced vasodilator effect in hypertrophic arteries is much closer to that in controls, as compared with SNP or labetalol. The increase in the EC_50_ for clevidipine (2-fold) to produce the same effect on both normal and hypertrophic arteries is less than that of SNP (3-fold) and labetalol (3.5-fold). These three drugs can all be used for the acute management of BP. Our results suggest that patients with vascular hypertrophy (usually shown in the late stage of hypertension) could be less resistant to clevidipine during acute BP control, as compared with the other drugs. The potent vasodilator effect of clevidipine could be partially explained by its molecular target, calcium channel, which is the common checkpoint for multiple factor-induced VSMC contraction. However, the cellular mechanisms under this phenomenon in VSMCs still need to be further investigated in the future.

## 4. Conclusions

Hypertrophic blood vessels require higher doses of antihypertensive drugs than normal or non-hypertrophic blood vessels to achieve the same vasodilator response. Different drugs have different profiles in relaxing the hypertrophic arterioles with clevidipine exhibited the lowest dose shift to relax the hypertrophic vessels compared to SNP and labetalol. The *in vitro* vasodilation may not be simply extrapolated to the clinical efficacy or outcome of each antihypertensive therapy; however, our data provide additional grounds for selecting specific antihypertensive drugs in clinical practice. Further clinical studies are needed to fully elucidate the use of different antihypertensive agents and clinical outcomes.
